# Intention as an indicator for subjective need: A new pathway in need assessment

**DOI:** 10.1186/1745-6673-5-20

**Published:** 2010-07-12

**Authors:** Uwe Rose, Linda Zimmermann, Ruth Pfeifer, Thomas Unterbrink, Joachim Bauer

**Affiliations:** 1 Department of Mental Health and Cognitive Capacity, Federal Institute for Occupational Safety and Health, Noeldnerstrasse 40-42, 10317 Berlin, Germany; 2 Department of Psychosomatic Medicine and Psychotherapy, University Medical Centre Freiburg, Hauptstr. 8, 79104 Freiburg, Germany

## Abstract

**Background:**

The current analyses focus on the need for services from the perspective of individuals considering preventive measures. A new approach imported from social and health psychology is used for assessing subjective need. This indicator is used for predicting actual health behaviour under field conditions and simultaneously other relevant background variables are taken into account.

**Methods:**

A mail survey was conducted prior to the start of a coaching program for teachers. A sample of *n *= 949 respondents were queried about mental distress and their intention to participate in the program. This intention to participate and actual attendance were taken as outcome variables in logistic regression analyses adjusted for relevant background variables.

**Results:**

Intention and participation in the coaching program three months later were associated with an unadjusted OR of 90.1 (95% CI: 39.2 - 207.0) for male teachers. For female teachers the crude effect was OR = 80.0 (95% CI: 45.7 - 140.1). The positive predictive value (PPV) was 96.4% among males and 94.5% among females. Adjusting for covariates results in higher values. Among female, but not among male teachers, the participation depended on psychological distress as assessed by the General Health Questionnaire (GHQ).

**Conclusions:**

There is strong evidence for using subjective need as an additional component in assessing the need for services and for predicting actual health behaviour. But it needs to be confined to intended behaviour which is under behavioural control.

## Background

A challenging task for providers and policymakers planning health care services and preventive measures lies in determining need for care. Neither a clear-cut definition nor operationalization exists and a variety of meanings grounded in different scientific views and perspectives [1] affect progress in need assessment and empirical analysis. Bradshaw's influential taxonomy of need [2] exemplifies this multi-perspective approach. According to Bradshaw normative need is defined by a standard given by an expert, professional, administrator or scientist. Felt need is equated with wants ("Do you feel that you are in need of...?"), expressed need represents the demand for a service, and comparative need is based on comparison of subpopulations receiving a service in question.

According to this taxonomy the main focus of current research is on normative need [3]: The case definition by a diagnosis or an ICD-10 classification act as a proxy for need when performing population-based surveys [4] aimed at determining prevalence. This definition and empirical procedure can be refined by combining caseness/prevalence with the assessment of disability/role impairment of "cases" [5,6].

The definition of need is also in scope of individual professionals (e.g. physician) or professional boards who define subjects in need for care and what kind of care is needed. In this case professionals as well as scientists take the availability of effective and cost-effective treatments or programs in health care into account; cases and non-cases are matched with available counteractive measures and supply in order to detect (un-)met need.

Evidence for effectiveness and efficiency of health care services indicates supply producing health gains or a "capacity to benefit" [7]. On top of this, it is a cornerstone for health economists who stress "the ability of people to benefit from health care provision" [8,9] in the light of scarce resources.

These opening comments, representing the view of scientific or professional experts, epitomize the *externally defined need for care*. This definition of need has to be distinguished from individuals' *subjective need for care *that reflects how individuals perceive their need and how need for care is defined by subjects themselves. If health care is provided the persons concerned might take on a perspective differing from that of experts. Hence, both perspectives may only overlap partially [10].

Felt need in the terminology of Bradshaw or perceived need actually used in surveys [11,12] refers to subjective need for care. Perceived need is assessed by using different types of questions: For instance, individuals are asked whether they think they needed help with emotional or mental health problems [11], whether they needed treatment [13], or what type of possible help they needed [14]. The responses to these different modes of questioning are indicators of an ambiguous concept [15] of perceived need. Furthermore, the theoretical integration of this concept into psychological theories (i.e., dealing with motivation and behaviour) is largely lacking and the mechanism for the formation of perceived need have not been elucidated. A theoretical and empirical underpinning of subjective need by current social or health psychology would be a fruitful starting point for formulating hypotheses and for developing scaling methods assessing subjective need. This issue is not merely an academic affair. The planning of health care supply also benefits from methods of need assessment that are indeed predictive for real demand and utilization.

### Subjective need for care or services

Externally defined need, as viewed by experts, takes the form of "person x or population x needs health service y in order to attain a certain health status level z". The subjective perspective of need is abbreviated by forms like "I need professional help". At this stage the kind of help and the goal might not be well elaborated and the individual's contemplation might be dominated more by a mixture of cognitions representing risk perception, the expectation of positive and negative consequences for different courses of actions or the expected capability to perform them. Asking subjects whether they feel or perceive a need addresses these considerations that precede health behaviour and actual usage of health care. Social and health psychology have a long tradition of dealing with these kinds of motivational factors and their influence on behaviour [16-18]. Based on this theoretical background the concept *intention *plays an important role as an antecedent of actual (health) behaviour. In empirical research intention is usually assessed by one-item questions; nevertheless it works as a powerful predictor for behaviour [19,20]. From this point of view the statement on individual's intention in a "yes" or "no" fashion serves as an indicator bridging the gap between a motivational process and real-life health behaviour. But the intention of doing something is closer to a point of decision compared to a state or process of vague need not necessarily resolved until then. Hence, this analysis focuses on intention as a proxy for subjective need for care or services. This proxy is well integrated in current research and it is assumed that intention is a good candidate for predicting actual service usage. The course of disease or disability status is not central to this kind of prognostic research. We are mainly concerned with the value of intention as a predictor of enrolment in and attendance of preventive programs. At this stage health behaviour is not severely restricted by a case of emergency or the burden of a disease. The hypotheses are that motivational factors, hereby represented by intention as proxy for subjective need, play a crucial role in predicting program attendance and actual health behaviour. It is assumed that health conditions in the domain of prevention are a weaker predictor than intention and that, on top of this, intention mediates the effect of health conditions on behaviour.

## Methods

### Design

This study is part of a larger prevention program called HEALTH PROMOTION FOR TEACHERS which was initiated and supervised by the Federal Institute of Occupational Safety and Health (FIOSH/BAuA). The program as a whole aimed at reducing stress in teachers through coaching, individualized guidance, and training programs at three different sites in Germany. One of these sites covered three school districts in South Western Germany around the city of Freiburg. Within these districts all 2,484 teachers in 19 grammar schools (Gymnasium) and 70 secondary modern schools (Hauptschule) were informed about a tailored coaching program available free of charge. Envelopes mailed out to teachers contained a covering letter, an application form and a questionnaire that teachers were asked to send back separately. The survey respondents constituted the mixed cohort at baseline t-1. The teachers were informed shortly about the program and procedures by means of a covering letter at baseline and by an informative meeting. The coaching program offered was free of charge but participation at five sessions during leisure time was required. The subset of volunteers applying for the coaching program were randomised to treatment and waiting control condition, and less than three month after the circular mailing the first session was held. The latter marks the starting point t0 of the coaching program or service use. The main focus of our prospective study is on the association between survey data at t-1 (mailing response) and service use at t0 less than three months later.

### Questionnaire and data

The questionnaire used in the survey at baseline (t-1) comprised questions regarding working conditions, occupational history and self-rating scales. With this questionnaire data on family background (having children, marital status) was obtained for reasons of adjustment. Part-time work was arbitrarily defined as working 75% or less of the predetermined workload. On top of this, additional information was given by two scales indexing (mental) health conditions: Psychological distress was assessed using German version of the 12-item General Health Questionnaire (GHQ-12) [21,22]. The GHQ-12 requires a recoding of negatively phrased items ("C-GHQ method") and a correction for possible response bias before a sum score is computed [23].

Emotional exhaustion was addressed by a mean value from a 9-item subscale from the Maslach Burnout Inventory (MBI-EE) in its German version (MBI-D) from Büssing and Perrar [24].

The main focus at baseline assessment was on subjective need. This was addressed by a single question "Do you intend to participate in a coaching course currently offered by us free of charge (see enclosed registration sheet)?" offering a "yes" or "no" answer. Actual behaviour approximately 3 months later (t0) is registered at the start of coaching group or of the waiting control condition.

### Analysis

Cross-sectional data from baseline (t-1) were the starting point for the first logistic regression analysis: The key variable "intention to participate" was regressed on other covariates at t-1 representing possible barriers or contributing factors. Covariates at t-1 are marital status, children at home and psychological distress, gauged by GHQ-12 and MBI-EE. The quantitative scales were transformed into terciles based on the score distribution of the total sample. Age was dichotomized at a cut-off value of 45 years and over. The logistic regression analysis was performed by testing different models including different subsets of covariates starting with simple models including background variables (abbreviated as M1). In model 2 (M2) "intention to participate" was cross-sectionally regressed onto these variables and onto an interaction of two background variables. In model 3 (M3) the outcome variable was regressed onto the background variables, interaction, and psychological distress.

After that, a similar modelling strategy was applied for a second regression analysis to predict actual behaviour at the starting point of treatment at t0. Therefore, service use as outcome was regressed in model M1 on the whole set of background variables from (t-1). Psychological distress was added in model M2 and in model M3 the outcome from the first regression analysis at t-1 was used as additional covariate.

The last analysis was focused on the association between health factors and participation. This result has to be taken into account for the discussion of intention as a possible mediator between health determinants and participation.

## Results

The sampling frame of *N *= 2484 included a higher proportion of female teachers (*n *= 1584; 63.8%) than male teachers (*n *= 900; 36.2%). Completed questionnaires were received from *n *= 949 (38.2%) with a response rates of 38% for female teachers and 37.7% for male teachers. Eight subjects gave no information about gender. Table 1 gives a sample description of the *n *= 949 respondents at t-1. The age distribution of both gender showed bimodality and a greater proportion of teachers 45 years or older (73.6% male teachers, 65.4% female teachers). Part-time work is more common in female (47.3%) than in male teachers (12.4%) and the disjunction of "single, divorced, and widowed" applied to 38.4% of the female teachers and 33.8% of the male teachers.

**Table 1:** description of sample at t-1

	Total sample	Male respondents	Female respondents
	*N *= 949	*n *= 339	*n *= 602
	*n *(%)	*n *(%)	*n *(%)
Age			
under 45	296 (31.7)	89 (26.4)	207 (34.6)
45 to 65	639 (68.3)	248 (73.6)	391 (65.4)
Marital status (MS)			
Married	623 (66.2)	252 (74.3)	371 (61.6)
Divorced	122 (13.0)	37 (10.9)	85 (14.1)
Widowed	14 (1.5)	2 (0.6)	12 (2.0)
Single	182 (19.3)	48 (14.2)	134 (22.3)
Children at home (CH)			
0	491 (52.9)	168 (50.0)	323 (54.6)
≥1	437 (47.1)	168 (50.0)	269 (45.4)
Work load			
Full time	614 (65.2)	297 (87.6)	317 (52.7)
Part time (≤3/4 of full load)	327 (34.8)	42 (12.4)	285 (47.3)
MBI-EE			
1^st ^tercile (≤2)	323 (34.4)	111 (32.7)	212 (35.4)
2^nd ^tercile (<2.8)	321 (34.2)	119 (35.1)	202 (33.7)
3^rd ^tercile (≤4.8)	294 (31.3)	109 (32.2)	185 (30.9)
GHQ-12			
1^st ^tercile (≤3)	326 (34.4)	123 (36.3)	203 (33.7)
2^nd ^tercile (≤5)	298 (31.4)	100 (29.5)	197 (32.7)
3^rd ^tercile (≤11)	308 (32.5)	113 (33.3)	194 (32.2)

number of teachers in each category (*n*), and the column percent (%); values may not add up due to missing values;

abbreviations: MS for marital status; CH for children at home,

MBI-EE for Maslach-Burnout-Inventory (Subscale Emotional Exhaustion)

GHQ-12 for General Health Questionnaire with correction for possible response bias

Table 2 gives an overview according to those teachers who intended to participate (outcome of first regression analysis) and who attend to the coaching program three month later (outcome of the second regression analysis). The percentages are conditioned on the categories of the background variables. This is supplemented by the crude association (OR) between background variables and both outcomes.

**Table 2:** description of intenders and participants based on covariates

male teachers	intenders (t-1)		participants (t0)	
	row %	OR (95% CI)	row %	OR (95% CI)
Age				
under 45	37.5	.5 (.3 - .8)	30.3	.6 (.3 - 1.0)
45 to 65	21.8		19.8	
Marital Status (MS)				
married	25.8	1.0 (.6 - 1.7)	24.1	1.1 (.6 - 2.0)
not married	25.6		21.8	
Children at home (CH)				
0	22.8	1.4 (.9 - 2.3)	20.2	1.3 (.8 - 2.1)
≥ 1	29.2		24.4	
Work load				
Full time	24.7	1.2 (.7 - 2.0)	20.4	1.5 (.9 - 2.5)
Part time	28.3		27.3	
MBI-EE				
1^st ^tercile (≤2)	18.9	1	16.2	1
2^nd ^tercile (<2.8)	25.4	1.5 (.8 - 2.7) 2^nd ^vs 1^st ^tercile	24.4	1.7 (.9 - 3.2) 2^nd ^vs 1^st ^tercile
3^rd ^tercile (≤4.8)	33.0	2.1 (1.1 - 3.9) 3^rd ^vs 1^st ^tercile	26.6	1.9 (1.0 - 3.6) 3^rd ^vs 1^st ^tercile
GHQ-12				
1^st ^tercile (≤3)	21.3	1	17.9	1
2^nd ^tercile (≤5)	28.0	1.4 (.8 - 2.7) 2^nd ^vs 1^st ^tercile	22	1.3 (.7 - 2.5) 2^nd ^vs 1^st ^tercile
3^rd ^tercile (≤11)	29.2	1.5 (.8 - 2.8) 3^rd ^vs 1^st ^tercile	28.3	1.8 (1.0 - 3.4) 3^rd ^vs 1^st ^tercile

divorced, widowed, single collapsed to one category				

**female teachers**	**intenders (t-1)**		**participants (t0)**	
	**row %**	**OR (95% CI)**	**row %**	**OR (95% CI)**
Age				
under 45	48.1	.6 (.4 -.8)	41.5	.7 (.5 - 1.0)
45 to 65	35.4		33	
Marital Status (MS)				
married	35.8	1.5 (1.1 - 2.2)	31.3	1.7 (1.2 - 2.4)
not married	46.3		43.7	
Children at home (CH)				
0	39.1	1.1 (.8 - 1.5)	37.5	.9 (.6 - 1.2)
≥ 1	40.9		34.6	
Work load				
Full time	38.3	1.1 (.8 - 1.6)	36.6	1.0 (.7 - 1.4)
Part time	40.5		35.8	
MBI-EE				
1^st ^tercile (≤2)	26.5	1	25.5	1
2^nd ^tercile (<2.8)	37.8	1.7 (1.1 - 2.6) 2^nd ^vs 1^st ^tercile	32.2	1.4 (.9 - 2.1) 2^nd ^vs 1^st ^tercile
3^rd ^tercile (≤4.8)	56.8	3.6 (2.4 - 5.5) 3^rd ^vs 1^st ^tercile	53.0	3.3 (2.1 - 5.0) 3^rd ^vs 1^st ^tercile
GHQ-12				
1^st ^tercile (≤3)	25.6	1	20.7	1
2^nd ^tercile (≤5)	42.6	2.2 (1.4 - 3.3) 2^nd ^vs 1^st ^tercile	38.1	2.4 (1.5 - 3.7) 2^nd ^vs 1^st ^tercile
3^rd ^tercile (≤11)	50.8	3.0 (2.0 - 4.6) 3^rd ^vs 1^st ^tercile	49.5	3.8 (2.4 - 5.8) 3^rd ^vs 1^st ^tercile

Table 3 shows the result for the cross-sectional analysis at t-1 stratified for gender.

**Table 3:** Logistic regression predicting intention (cross-sectional)

Male teachers (n = 339)			
	model M1	model M2	model M3
**Variables included**	**OR (95% CI)**	**OR (95% CI)**	**OR (95% CI)**

Age (45+)	.5 (.3 -.8)	.5 (.3 -.8)	.4 (.2 -.7)
Marital status (MS)	.9 (.5 - 1.7)	.6 (.3 - 1.3)	. 6 (.3 - 1.3)
Children at home (CH)	1.3 (.8 - 2.3)	1.00 (.6 - 1.8)	1.0 (.6 - 1.9)
MS X CH^1^		.3 (.1-1.0)	.3 (.1 - 1.1)
GHQ-12 (1/2)			1.2 (.6 - 2.3)
(1/3)			1.1 (.5 - 2.3)
MBI-EE (1/2)			1.4 (.7 - 2.7)
(1/3)			2.2 (1.0 - 4.7)
	-2LL: 371.7	-2LL: 367.9	-2LL: 361.0
	Δ CHI^2^: 9.0	Δ CHI^2^: 3.9	Δ CHI^2^: 6.9
	p = .03 (df = 3)	p = .05 (df = 1)	p = .14 (df = 4)
Nagelkerkes R^2^	.04	.06	.09
Hosmer-Lemeshow-Test	CHI^2^: 4.2	CHI^2^: .1	CHI^2^: 13.5
	p = .52 (df = 5)	p = .99 (df = 4)	p = .09 (df = 8)

**Female teachers (n = 602)**			
**Variables included**	**OR (95% CI)**	**OR (95% CI)**	**OR (95% CI)**

Age (45+)	.7 (.5 - .9)	.7 (.5 - .9)	.6 (.4 - .8)
Marital status (MS)	1.4 (1.0 - 2.1)	1.4 (.6 - 2.2)	1.2 (.7 - 1.9)
Children at home (CH)	1.6 (.9 - 1.8)	1.2 (.8 - 1.9)	1.2 (.8 - 2.0)
MS X CH^1^		.9 (.4 - 1.9)	.8 (.4 - 1.7)
GHQ-12 (1/2)			1.8 (1.1 - 2.8)
(1/3)			2.0 (1.2 - 3.3)
MBI-EE (1/2)			1.3 (.8 - 2.0)
(1/3)			2.6 (1.5 - 4.3)
	-2LL: 764.3	-2LL: 764.2	-2LL: 720.17
	Δ CHI^2^: 12.1	Δ CHI^2^: .1	Δ CHI^2^: 44.0
	p = .01 (df = 3)	p = .72 (df = 1)	p < .00 (df = 4)
Nagelkerkes R^2^	.03	.03	.12
Hosmer-Lemeshow-Test	CHI^2^: 2.4	CHI^2^: 2.1	CHI^2^: 5.2
	p = .67 (df = 4)	p = .83(df = 5)	p = .74 (df = 8)

^1 ^MS X CH: Interaction effect of marital status and children at home

In M1 the odds for forming an intention was reduced for older male teachers (OR = .5; 95% CI:.3 - .8) compared to the younger male teachers. There were no main effects for marital status or having children at home on forming an intention, but the effect for interaction was near at the significance level (M2; OR = .3; 95% CI: .1 - 1.0). Psychological distress indexed by GHQ-12 and MBI was not associated with the intention to participate.

For female teachers age also had a negative effect on intention (M1; OR = .7; 95% CI: .5 - .9). There were no effects for marital status, children at home or interaction. But in contrast to male teachers psychological distress assessed by the GHQ-12 was associated with the intention to participate. Changing from the first to the second tercile of the GHQ-12 distribution raised the odd in M3 for an intention by a factor of OR = 1.8 (95% CI: 1.1 - 2.8). For MBI-EE in M3 there was only a statistical significance between the first and third tercile (OR = 2.6; 95% CI: 1.5 - 4.3) according intention as outcome variable.

Table 4 shows the result for predicting participation three months later. Age was negatively associated with participation for male teachers in M2 (OR = .5; 95% CI: .3 - .9). Statistically, the effects for children at home, marital status and scales for psychological distress on participation at t0 were not significant. And a significant interaction between marital status and children at home was observed for the male teachers (M1; OR = .2; 95% CI:.1 - .9). Controlling for covariates mentioned before, there was a strong association between pre-tested intention and participation in the coaching program (OR = 121.1; 95% CI: 46.1 - 318.2).

**Table 4:** Logistic regression predicting participation (longitudinal)

Male teachers (n = 339)			
	model M1	model M2	model M3
**Variables included**	**OR (95% CI)**	**OR (95% CI)**	**OR (95% CI)**

Age (45+)	.5 (.3 - 1.0)	.5 (.3 - .9)	.9 (.4 - 2.4)
Marital status (MS)	.7 (.3 - 1.5)	.6 (.3 - 1.4)	.9 (.2 - 3.3)
Children at home (CH)	.9 (.5 - 1.7)	.9 (.5 - 1.7)	.7 (.2 - 1.8)
MS X CH^1^	.2 (.1 - .9)	.3 (.1 - 1.0)	.3 (.0 - 2.8)
GHQ-12 (1/2)		1.2 (.6 - 2.4)	1.2 (.4 - 3.9)
(1/3)		1.6 (.8 - 3.4)	3.0 (.8 - 11.0)
MBI-EE (1/2)		1.6 (.8 - 3.2)	1.6 (.5 - 4.9)
(1/3)		1.6 (.7 - 3.5)	.6 (.2 - 2.3)
Intention (t-1)			121.1 (46.1 - 318.2)
	-2LL: 344.1	-2LL: 337.9	-2LL: 156.7
	Δ CHI^2^: 9.7	Δ CHI^2^: 6.2	Δ CHI^2^: 181.2
	p = .05 (df = 4)	p = .19 (df = 4)	p < .01 (df = 1)
Nagelkerkes R^2^	.04	.07	.68
Hosmer-Lemeshow-Test	CHI^2^: .9	CHI^2^: 3.4	CHI^2^:4.9
	p = .92 (df = 4)	p = .91 (df = 8)	p = .76 (df = 8)

**Female teachers (n = 602)**			
**Variables included**	**OR (95% CI)**	**OR (95% CI)**	**OR (95% CI)**

Age (45+)	.8 (.5 - 1.1)	.7 (.5 - 1.0)	1.1 (.6 - 2.1)
Marital status (MS)	1.5 (.9 - 2.4)	1.3 (.8 - 2.2)	1.6 (.7 - 3.5)
Children at home (CH)	1.0 (.6 - 1.5)	1.0 (.6 - 1.6)	.7 (.3 - 1.4)
MS X CH^1^	.9 (.4 - 2.0)	.8 (.4 - 1.9)	1.0 (.3 - 3.4)-
GHQ-12 (1/2)		2.1 (1.3 - 3.4)	2.0 (1.0 - 4.2)
(1/3)		2.9 (1.7 - 4.9)	3.7 (1.6 - 8.5)
MBI-EE (1/2)		.9 (.6 - 1.5)	.6 (.3 - 1.2)
(1/3)		1.8 (1.1 - 3.0)	.7 (.3 - 1.5)
Intention			85.7 (46.0 - 159.6)
	-2LL: 746.9	-2LL: 700.4	-2LL: 346.8
	Δ CHI^2^: 9.2	Δ CHI^2^: 46.5	Δ CHI^2^: 353.6
	p = .06 (df = 4)	p < .00 (df = 4)	p < .00 (df = 1)
Nagelkerkes R^2^	.02	.13	.70
Hosmer-Lemeshow-Test	CHI^2^: 1.3	CHI^2^: 2.3	CHI^2^: 12.1
	p = .94 (df = 5)	p = .97 (df = 8)	p = .15 (df = 8)

^1 ^MS X CH: Interaction effect of marital status and children at home

Psychological distress among female teachers indexed by GHQ-12 was a significant predictor for participation with an OR = 2.1 (M2; 95% CI: 1.3 - 3.4) by comparing the first and second tercile of GHQ-12. According the difference between the first and third tercile of GHQ-12 in model M2 participation was predicted by an OR = 2.9 (95% CI: 1.7 - 4.9). Only the highest tercile of the MBI-EE showed higher odds for participation compared to the lowest tercile (M2; OR = 1.8; 95% CI: 1.1 - 3.0). The difference between first and second MBI-EE tercile did not reach significance (M2; OR = .9; 95% CI: .6 - 1.5). Again, there was a strong association between intention and participation when controlled for the other covariates (M3; OR = 85.7; 95% CI: 46.0 - 159.6).

The cross-tabulation table 5 displays the crude effect of intention not adjusted by other covariates. A proportion of 96.4% of male teachers who reported their intention in the survey participated in the coaching program. A smaller predictive value of 77% was found for predicting non participation of male teachers. In this group intention and participation are associated by an OR = 90.1 (95% CI: 39.2 - 207.0). For female teachers the crude effect was OR = 80.0 (95% CI: 45.7 - 140.1) with a predicted valid proportion of 94.5% participants among intenders and 82.4% non-participants among non-intenders.

**Table 5:** cross tabulation of intention (t-1) and participation (t0)

**Male teachers**		**participation (t0)**			**Female teachers**	**participation (t0)**		
		**(+)**	**(-)**			**(+)**	**(-)**	
		
Intention at t-1	+	242	9	251		341	20	361
								
	-	20	67	87		42	197	239
						
		262	76	338		383	217	600
				
PV+ | PV-		96.4%	77.0%			94.5%	82.4%	

The results of the last analysis focused on the association between psychological distress at t-1 and participation at t0 controlled for age, marital status and children at home. Compared to the first tercile of MBI-EE (t-1) as reference category the odds for participation of male teachers raised within the third tercile of MBI-EE by a factor of OR = 2.1 (95% CI: 1.0 - 4.1) and for female teachers by an OR = 3.1 (95% CI: 2.0 - 4.8).

For male teachers an association between GHQ-12 and participation was found by comparing the first tercile of GHQ-12 as reference category with the third tercile (OR = 2; 95% CI: 1.1 - 3.8). For female teachers higher odds for participation were found both for the third tercile (OR = 3.8; 95% CI: 2.5 - 6.0) and for the second tercile (OR = 2.4; 95% CI: 1.5 - 3.6) compared to the first tercile of GHQ-12 as reference category.

## Discussion

Increasing age seems to be a barrier for forming an intention, both among female and male teachers. But only for male teachers actual participation in the coaching program is affected by age. Based on our own hypothesis, we expected children at home or marital status to be time constraints working as a barrier, especially for female teachers. But prima facie our results did not support this assumption. A closer inspection of tabulated data for unmarried *male *teachers revealed that having children at home rather *promotes *forming an intention. The non-significance in the case of female teachers does not mean that time restrictions and family background have no effects at all. The interpretation is simply hampered by the high proportion of female teachers working part-time because of the family background. This has to be taken into account as a possible counteracting effect masking the effect of family and children at home for female teachers.

The coaching program, that was offered to 2,484 teachers aimed at reducing psychological distress or stress reactions. The results showed that an increase in scores for GHQ-12 or MBI-EE tended to go along with an intention to participate. Hence, psychological distress had a positive effect on the motivation to use programs tailored to alleviate it. This was best illustrated by the association between GHQ-12 and intention among the 602 female teachers and for the highest tercile of the MBI-EE. The data from the smaller group of 339 male teachers and the hence less powerful analysis yielded no significant results. The regression analysis based on participation as outcome variable showed a similar pattern of results. Psychological distress among female teachers indicated by GHQ-12 and MBI-EE was associated with participation in the coaching program and this was accompanied by stronger effects for GHQ-12 than for MBI-EE.

The regression analysis applied to model 2 and model 3 highlighted the role of intention as a predictor variable. Within the set of covariates in model 3 only intention contributed to predicting actual behaviour three months later. Additionally, the crude association between the single predictor intention and participation as a behavioural indicator showed high values. On top of this, these odds ratios increase after including intention and control variables within the same set of covariates in model 3. This likely indicates a suppressor effect resulting from the combination of intention and other covariates and the elimination of irrelevant variation. Yet, improving the predictive power by using the full set of covariates comprising intention runs into problems of instability. Parameter estimates were characterized by broader confidence intervals while the goodness-of-fit of the model decreased. Thus, for reasons of stability and efficiency there are good reasons for disregarding other covariates and suppressor effects and to rely primarily on intention as the main variable for the purpose of simple prediction.

One comment has to be made on the hypothesis of mediation: There might be an effect of psychological distress - measured by MBI or GHQ - on participation which is mediated by intention as intervening or process variable. The final results in the former section show that there is an effect of psychological distress on participation that may be mediated by intention. Table 3 also shows that there is an association between psychological distress and intention and table 4 reports the association between intention and participation. But as shown in table 4 the association between distress and participation adjusted for other covariates is higher than zero. Rather there is a substantial association between distress and participation even though intention has been taken into the equation. These data do not support a hypothesis of complete mediation of distress by intention but the results are also consistent with a partial mediation. Even for the crude association between intention and participation a high odds ratio value was returned. Attitude research which explores the association between motivational factors and behaviour in the field of social or health psychology provides a tentative explanation for the magnitude of this effect. According to this research asking for a specific behaviour is linked to four aspects: (a) a specific action or behaviour, (b) performed toward a target, (c) in a context, (d) at a time or occasion. This is partly mirrored by the current study and by asking subjects for participation (a) in a coaching course (b) currently offered free of charge (c). An exact definition for time or occasion (d) was not provided in this study. According to the principle of compatibility [25] maximally strong relations between attitudes and behaviours are expected, if action, target, context, and time elements are assessed at the same level of generality or specificity. This matching was realized by the single item phrase aiming at intention that corresponds to the specific single behaviour three months later. Furthermore, the time interval between the assessment of intention and behaviour was minimised and this fact also contributes to a high degree of association.

The intention to act in a specific way earmarks a cumulative endpoint of a motivational process that follows from considering one's own health condition, positive and negative consequences for different courses of (non-)action, self-efficacy and the perception of possible barriers. All these important determinants contribute to/enter into a decision to act and hence intention becomes a very powerful tool for need assessment and the prediction of service use. Theories and results of health and social psychology from the last decades [16-18] pointed to these relevant determinants (e.g. risk perception, expectancies of consequences and self-efficacy, etc.). Hence, knowledge from these sciences helps to understand the contributing factors for individuals to use preventive measures or health services even if externally defined need (e.g. burden of disease) is not evident. It is not sufficient to offer services without knowing the motivational or volitional factors in the target group relevant for service use. Successful implementation of programs, usage and compliance depends heavily on tailored services which meet the subjective need of users. A weakness of the current study results from the sampling procedure. The first wave of respondents does not constitute a representative sample of teachers in these school districts. It can safely be assumed that respondents in the first wave are more interested in the topics of the questionnaire than non-respondents. On top of this, this bias introduced by a selection of "interested" teachers might be associated with the predictor intention and actual coaching attendance three months later. This kind of bias is not confined to the current study. This probably is a general problem for need assessments that are based on subjective need. As a consequence of this we expect stronger associations between intention and participation for the selection of interested teachers (32%). But this relation cannot be assumed for the remaining 68% of non interested teachers. The external validity of our results or the generalisability depends heavily on the attributes of the sample used for assessment and the sampling process. But little information according the population was given and therefore drawbacks have to be made with caution. Only the distribution for gender within the population was given. But an indication of a bias caused by a differential response rate of male and female teachers was not evident.

In addition to methodological constraints there are other reasons not to rely on intention as the sole indicator of subjective need. These are rooted in restrictions imposed on application: We presume that participation in the coaching program in this study reflects a behaviour which is predominantly under volitional control and that using intention as a predictor is only useful in this kind of setting. We also expected intention to be a weaker predictor of future behaviour in the sense that the behaviour performed is not a product of choice (i.e. volitional). This is exemplified by subjects who act in a relatively spontaneous or impulsive way, without forming an explicit intention beforehand. Another example is given by severe injuries caused by an accident when the subjective need of the victim is heavily determined by the basic need to survive and therefore the decision to use medical services is not or only partly under volitional control. Some behaviour requires special skills and abilities, support from or cooperation of others, resources (money, time etc.) or just the opportunity to act (i.e. a tailored supply and time frame). Asking subjects about their intention to make use of a service when - i.e. monetary - resources required are not available exemplifies a behaviour not being under control.

A further restriction concerns the conceptualization of intention. In the current study the subjects were not asked for vaguely formulated wishes or broad intentions. Rather, subjects were asked for a clear *plan *to engage in a single behaviour. This might be a very extensive interpretation of intention as a concept. But moving in the continuum from mere wishes to detailed plans and actual behaviour provides a better basis for the prediction of health-relevant demand and usage.

## Conclusions

The restrictions mentioned above are strong arguments against relying solely on intention as an indicator of need. The option recommended here is to use it *in addition *to the traditional approach. Asking for intention is a very simple and most efficient procedure according to the prediction of health relevant behaviour being under behavioural control. In this context of application the assessment of subjective need of the target population gives the basis for realistically planning and organizing public health services and for optimizing the supply.

## Competing interests

The authors declare that they have no competing interests.

## Authors' contributions

UR participated in the design of the study and performed the statistical analysis. He also drafted the manuscript.

LZ participated at the design of the study and at the data acquisition followed by data preparation for the current analysis.

RP participated at data acquisition and data preparation for the current analysis.

TU participated in the design of the study and at the data preparation.

JB participated at the coordination of the current study and helped to draft the manuscript.

All authors read and approved the final manuscript.
